# A rare variant of lichen planopilaris: Familial Graham-Little-Piccardi-Lassueur syndrome in Saudi Arabia

**DOI:** 10.1016/j.jdcr.2026.02.001

**Published:** 2026-02-06

**Authors:** Sadeen T. Shahbar, Hadeel A. Maaddawi, Yara E. Aljefri, Nawal R. Al Yamani, Zainab A. Khalaf, Mostafa A. Safar

**Affiliations:** a Department of Dermatology, Makkah Health Cluster, Makkah, Saudi Arabia; b Department of Dermatology, College of Medicine, King Saud University, Riyadh, Saudi Arabia; c Department of Dermatology, Jeddah Health Cluster, Jeddah, Saudi Arabia; d Department of Dermatology, King Fahad General Hospital, Jeddah, Saudi Arabia; e Department of Family Medicine, Jeddah First Health Cluster, Jeddah, Saudi Arabia; f College of Medicine, King Abdulaziz University, Jeddah, Saudi Arabia

**Keywords:** Alopecia, Graham-Little-Piccardi-Lassueur syndrome, Lichen Planopilaris

## Case description

**Fig 1:**
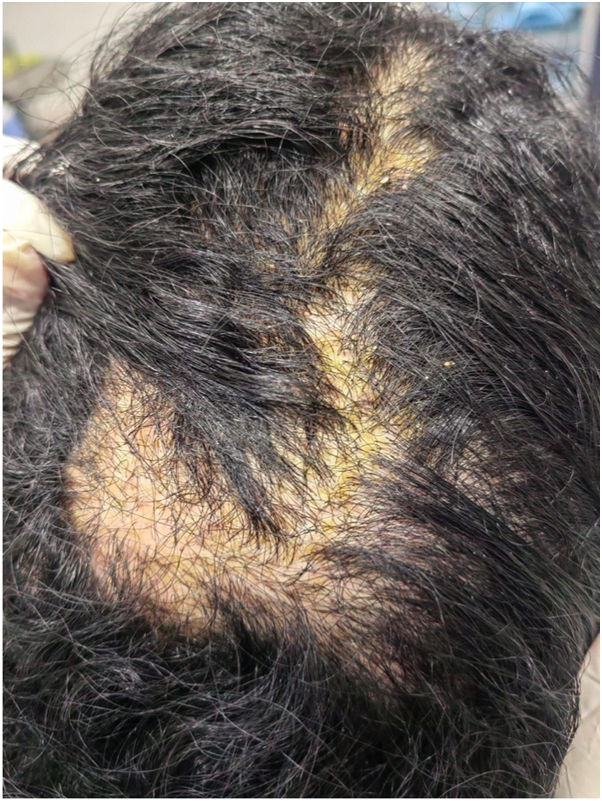
Patchy areas of cicatricial alopecia involving the parietal scalp, demonstrating perifollicular scale and erythema, and loss of follicular ostia, consistent with LPP.

**Fig 2:**
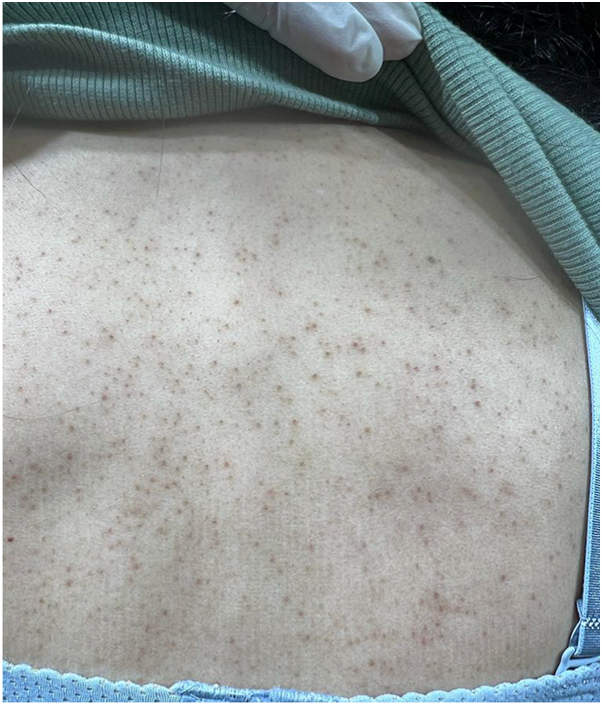
Diffuse follicular keratotic papules involving the upper back of the index female patient, consistent with the extracephalic cutaneous manifestations of Graham-Little-Piccardi-Lassueur syndrome.

**Fig 3:**
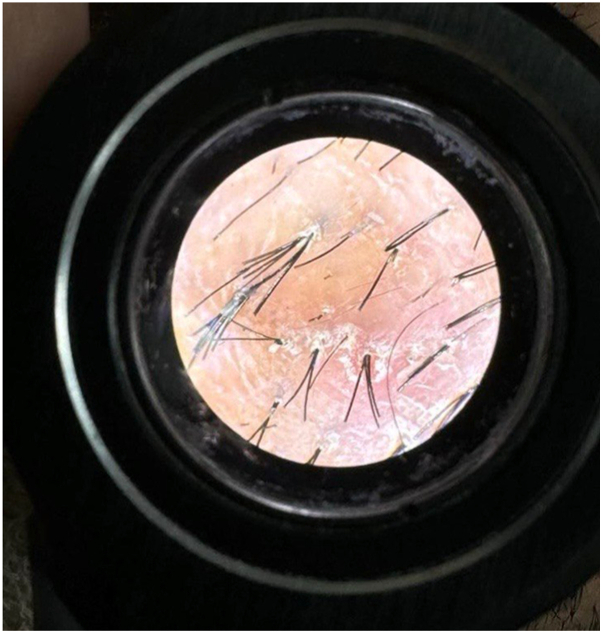
Dermoscopy findings suggestive of the trichoscopic features of lichen planopilaris with perifollicular scaling and erythema and absent follicular openings.

A 28-year-old woman from Jeddah, Saudi Arabia, presented with a 1-year history of progressive scalp hair loss associated with pruritus and scalp tenderness. Hair loss initially involved the parietal region and gradually progressed to affect the entire scalp. She additionally reported a 14-year history of widespread follicular papules, first noted on the upper extremities and later involving the trunk and lower limbs. Cutaneous examination revealed patchy cicatricial alopecia of the scalp with associated hyperpigmented, keratotic follicular papules distributed over the scalp, trunk, and extremities (Figs 1 and 2). The eyebrows, axillae, and pubic regions were spared. Trichoscopic evaluation demonstrated perifollicular erythema, scaling, and absence of follicular openings, findings consistent with lichen planopilaris (LPP) (Fig 3). Scalp biopsy showed nonspecific features of cicatricial alopecia and was performed primarily to exclude histologic mimickers rather than to confirm the diagnosis.

**Fig 4:**
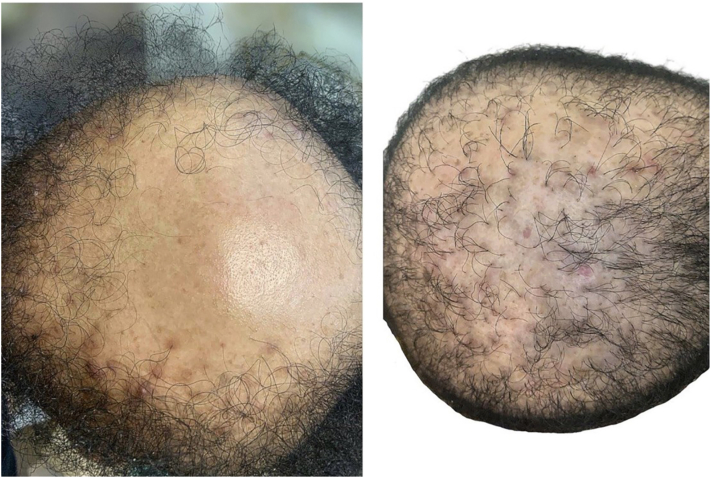
Scalp involvement in affected siblings demonstrating alopecia with a similar distribution pattern, supporting a familial presentation.

Family history was significant for similar cutaneous and scalp findings in her father and 2 brothers, all of whom developed follicular papules followed by patterned scalp alopecia during adolescence or early adulthood, supporting a familial form of disease (Fig 4). One affected brother failed to respond to hydroxychloroquine therapy, while the other remained untreated.

The patient was treated with oral isotretinoin (20 mg daily for 6 months), resulting in approximately 80% improvement of follicular papules. Hydroxychloroquine (200 mg daily for 2 years) led to stabilization of hair loss without regrowth. Intralesional corticosteroids provided symptomatic relief but did not induce hair regrowth, and a 6-month trial of dutasteride (0.5 mg daily) yielded no clinical benefit.

**Question: A 28-year-old woman presents with progressive scarring alopecia of the scalp associated with pruritus and pain. She has a long-standing history of widespread follicular papules on the trunk and extremities. Trichoscopy reveals perifollicular erythema, scaling, and absent follicular openings. Family history is notable for similar findings in her father and 2 brothers. Which of the following is the most likely diagnosis?**

**A.** Graham-Little-Piccardi-Lassueur syndrome
**B.** Classic lichen planopilaris
**C.** Folliculotropic mycosis fungoides
**D.** Familial keratosis pilaris with coincidental alopecia
**E.** Central centrifugal cicatricial alopecia

**Correct Answer: A. Graham-Little-Piccardi-Lassueur** **syndrome.**

## Answer discussion

This presentation is most consistent with Graham-Little-Piccardi-Lassueur syndrome (GLPLS), a rare variant of lichen planopilaris characterized by the triad of cicatricial scalp alopecia, widespread follicular keratotic papules, and variable non-scarring alopecia at other sites. The latter feature, commonly involving the axillary or pubic regions, may be absent or delayed, particularly early in the disease course.^1^ The prominent extracephalic follicular papules and strong familial clustering favor GLPLS over classic lichen planopilaris, which typically lacks these features. Familial cases of GLPLS have been reported and suggest an underlying genetic predisposition.^2^, ^3^, ^4^, ^5^ Folliculotropic mycosis fungoides would demonstrate atypical lymphocytes on histopathology, keratosis pilaris does not result in scarring alopecia, and central centrifugal cicatricial alopecia predominantly affects women of African descent with vertex-centered involvement.^1^

In this case, the diagnosis was established primarily on clinical and trichoscopic findings. Histopathologic features in GLPLS are frequently nonspecific and are most useful for excluding alternative diagnoses rather than confirming the condition.

## Conflicts of interest

None disclosed.
